# Effect of Informed Conditions on Sensory Expectations and Actual Perceptions: A Case of Chocolate Brownies Containing Edible-Cricket Protein

**DOI:** 10.3390/foods10071480

**Published:** 2021-06-25

**Authors:** Cristhiam E. Gurdian, Damir D. Torrico, Bin Li, Georgianna Tuuri, Witoon Prinyawiwatkul

**Affiliations:** 1 School of Nutrition and Food Sciences, Louisiana State University Agricultural Center, Baton Rouge, LA 70803, USA; cgurdi3@lsu.edu (C.E.G.); gtuuri@agcenter.lsu.edu (G.T.); 2 Department of Wine, Food and Molecular Biosciences, Faculty of Agriculture and Life Sciences, Lincoln University, Lincoln 7647, New Zealand; damir.torrico@lincoln.ac.nz; 3 Department of Experimental Statistics, Louisiana State University Agricultural Center, Baton Rouge, LA 70803, USA; bli@lsu.edu

**Keywords:** alternative insect protein, cognitive cue, consumer behavior, entomophagy, insect consumption

## Abstract

Currently, many consumers are reluctant to consume edible-cricket protein (ECP). Chocolate brownie (CB) formulations without (WO) and with (W) 6%*w*/*w* ECP (CBWO and CBW, respectively) were presented under two informed conditions: formulated without ECP (ECP−) and formulated with ECP+benefits (ECP+). CBWO− (CBWO presented with the “ECP−” claim), CBWO+ (CBWO presented with the “ECP+” claim), CBW− (CBW presented with the “ECP−” claim), and CBW+ (CBW presented with the “ECP+” claim) were evaluated by 210 consumers for expected and actual attribute liking, and after-tasting consumption and purchase intent. Multi-way ANOVA, principal component analysis, and agglomerative clustering examined liking. Cochran-Q tests compared actual-liking profiles, purchase and consumption intent. Before tasting, CBW− obtained the lowest appearance liking, flavor liking was higher for ECP− than for ECP+ for either formulation, and ECP+ decreased aroma and overall liking only for CBWO. After tasting, CBWO had higher liking than CBW (except for aroma) for either informed condition. Regardless of the formulation, ECP− and ECP+ had similar actual liking. Nevertheless, ECP+ prevented negative disconfirmation for both formulations while ECP− decreased texture liking (for CBWO) and all liking (for CBW) upon tasting. Females’ consumption intent was higher for CBWO regardless of the informed condition, but CBW+ achieved a similar purchase intent to CBWO− for both genders.

## 1. Introduction

One billion people presently experience inadequate protein intake and protein-energy malnutrition, resulting in impaired growth, development, and health. The world population is expected to reach 9.6 billion by 2050, translating into a 70% increase in the actual global food demand. Hence, the development of sustainable and nutrient-dense foods from alternative sources is needed to overcome food insecurity [1]. While food insecurity is the main concern in developing countries given the projected increase in the global population, for developed countries such as the United States, food insecurity is rather a minor issue. Instead, for industrialized countries, food safety and environmental sustainability of the food production system are the main topics driving the current and future health problems associated with foods [2].

The animal-based food production system requires more environmental resources and generates more pollution than edible-insect production [3]. Incorporation of edible insects rich in protein, such as crickets, into human diets, can offer sustainable alternatives to conventional protein sources, because insects are more efficient in the body-mass conversion of feeding [4].

About 2000 species of insects are edible, but only around 113 countries worldwide practice entomophagy, or the eating of insects [5]. The nutritional profile and quality of insects depend on various factors including the species, developmental stage, origin, and diet, etc. [6]. The protein content in edible insects ranges from 35–61% on a dry basis [7], surpassing the protein content [2] of popular plant-derived sources (e.g., soybeans, beans, lentils), and making it comparable to that of conventional high-quality animal-derived sources [8,9,10].

Rumpold and Schlüter [7] reported that members of the Orthoptera species had superior protein content (upper range of 77.13% dry basis) compared to the maximum protein content observed in plants (35.8% dry basis in soybeans). A feeding trial evaluating crickets’ protein quality found equal or improved amino acid content of the cricket meals compared to soy protein [11]. Köhler et al. [12] found that Bombay locust, scarab beetle, house cricket, and mulberry silkworm from Thailand were all high in protein. Oibiokpa et al. [13] assessed the protein quality of moth caterpillar, termite, cricket, and grasshopper in rat diets, and reported that crickets had the highest amino acid score (0.91), protein efficiency ratio (PER; 1.78), net protein ratio (3.04), biological value (93.02%), and protein digestibility corrected for amino acid score (0.73). Crickets were superior to casein in terms of PER (1.78 vs. 0.86) and biological value (93.02 vs. 73.45) and similar in net protein ratio (3.04 vs. 2.74) and NPU (75.20 vs. 72.42), but had lower true digestibility (80.82 vs. 98.19), respectively.

Edible insects are also rich (10–60% dry basis) in fat and lipids [4,14] with a lower omega 6:omega 3 ratio. Most edible insects are good sources of energy, essential amino acids, trace elements, and minerals providing B-complex vitamins [14].

Despite the nutritional and environmental advantages of entomophagy, there is still a significant aversion to insects as foods [15,16] associated with food neophobia, poorly perceived sensory quality, or negative product-elicited emotions mainly in the Western world [17]. Efforts to change attitudes have resulted in food products yielding poor sensory-liking and adverse emotional reactions [18]. However, consumers’ preferences may be altered over time through repeated exposure in which the social, religious, and cultural environments play a crucial role depending on age, gender, education, economic status, and degree of health consciousness [19].

The consumption of edible insects in Western culture is very limited [20]. Food safety concerns (microbial and chemical health risks) are among the main obstacles for introducing entomophagy to Western diets even for consumers’ willingness to consume edible insects and/or edible insect ingredients [9]. However, previous research studying the perception of insect ingredients among Westerners has shown that depending on the product sensory profile [21], consumers may be able to repeat insect consumption if they enjoyed a previous eating experience. Moreover, hedonic claims [22] and informative sessions regarding edible insects (including their safety regulations and benefits) [23] seemed to improve familiarity and alleviate negative preconceptions about entomophagy. Fischer and Steenbekkers [24] reported that Westerners were more receptive to crickets, mealworms, and grasshoppers than other edible insects. On the other hand, Ardoin and Prinyawiwatkul [15] found that U.S. consumers were more willing to try insect ingredients in protein/energy bars, chips/snack crackers and protein shakes, but bakery/cereal products and snacks/candy also obtained positive willingness to try by over 50% of the survey respondents. In their study, unfamiliarity with edible insects was the most limiting factor for the willingness to consume insects, but communicating benefits derived from entomophagy positively impacted the willingness to try for all products.

The science behind edible insects is still pioneering in the Western world. The development of acceptable products containing insect proteins could be achieved with a thorough understanding of consumers’ expectations, needs, sentiments, and drivers of liking for this market niche. Introducing edible-cricket protein as a processed ingredient [6] in a familiar product [21,25], such as chocolate brownies, while providing information about the sustainability and health benefits of entomophagy may alleviate consumer reluctance to consume edible insects.

To our knowledge, the combined effect of providing or withholding information about the presence and benefits (sustainability + nutritional quality) of ECP on consumer acceptability, willingness to consume and to purchase chocolate brownies with and without ECP has not been studied. Hence, this study compared the sensory acceptability, consumption intent, and purchase intent of chocolate brownies without (CBWO) and with 6% ECP (CBW) when presented under two informed conditions regarding the absence (ECP−) or presence of ECP and its environmental and nutritional quality associated benefits (ECP+).

## 2. Materials and Methods

### 2.1. Chocolate Brownie Samples Preparation

Betty Crocker fudge chocolate brownie batter mixes (General Mills Sales, Inc., Minneapolis, MN, USA) containing sugar, enriched bleached flour (wheat flour, niacin, iron, thiamin mononitrate, riboflavin, folic acid), cocoa processed with alkali, palm oil, corn syrup, corn starch and 2% or less of: carob powder, salt, canola oil, and artificial flavor, USDA grade A large-white eggs (Great Value, Walmart Stores, Inc., Bentonville, AR, USA), and Wesson canola oil (Conagra Brands, Chicago, IL, USA) were purchased at Walmart Supercenter (Baton Rouge, LA, USA). Griopro edible cricket protein (ECP) powder (All Things Bugs LLC, Midwest City, OK, USA) made of whole crickets (*Acheta domesticus* and *Gryllodes sigillatus*) was purchased online from www.cricketpowder.com (accessed on 28 January 2019). Batches of each chocolate brownie formulation (without ECP, CBWO, and with 6% *w*/*w* ECP, CBW) were prepared following the cooking instructions provided on the batter mix and scaled up to provide sufficient samples for the consumer test. The 6% *w*/*w* ECP concentration was selected based on a preliminary trial with 25 subjects. A range of concentrations (3–10% *w*/*w*) was tested for which 6% *w*/*w* yielded a recognizable difference compared to control (0% ECP) in half of the subjects but without largely compromising the sensory acceptability or identifying a particular sensory characteristic that would reveal the identity of the ingredient. For each batch, eggs (875 g), water (258 g), canola oil (621 g), and batter mix (3128 g) were stirred together in a Globe SP20 commercial food mixer (Globe Food Equipment CO, Dayton, OH, USA) at speed 2. For CBW, ECP (312 g) was additionally mixed with the other ingredients. The mixture was then poured into a 45.7 cm × 66 cm aluminum pan and baked in a pre-heated OV310G mini rotating rack oven (Baxter Mfg, a Division of ITW FEG, LLC, Orting, WA, USA) at 325° F for 52 min. Batches from both formulations were stored separately at room temperature in sealed plastic containers overnight until the analyses and consumer test.

### 2.2. Sensory Evaluation

#### 2.2.1. Subjects

Untrained subjects 18 years of age and older (*n* = 210, 98 males and 112 females) were recruited at Louisiana State University (LSU), Baton Rouge, Louisiana, USA, and screened for: (1) willingness to evaluate test samples that contain edible-cricket protein (ECP) powder, (2) self-report on no allergies or adverse reactions to the test samples, (3) not having impaired vision/color blindness or taste/smell disorders that would compromise their sensory evaluations, and (4) being regular consumers (at least once per month) of chocolate brownies based on self-reported responses. All participants were informed of any allergens present in the test samples. Subsequently, subjects agreed with and signed a consent form included in the research protocol approved (IRB # HE 18-9 and # HE 18-22) by the LSU Agricultural Center Institutional Review Board.

#### 2.2.2. Consumer Test

On the day of the study, chocolate-brownie samples were cut into 3 cm cubes and placed in 2 oz. clear plastic-lidded cups labeled with three-digit blinding codes. Each panelist evaluated all four treatments (before and after tasting) in one session, cleansing their palate with unsalted crackers and water before the first sample and in between samples (when instructed to taste). The consumer test took place in partition booths equipped with white lights in the Sensory Services Laboratory at LSU under a controlled environment (ca. 25 °C). After the evaluation, participants were compensated with soft drinks and/or brewed coffee.

#### 2.2.3. Questionnaire

**Figure 1:**
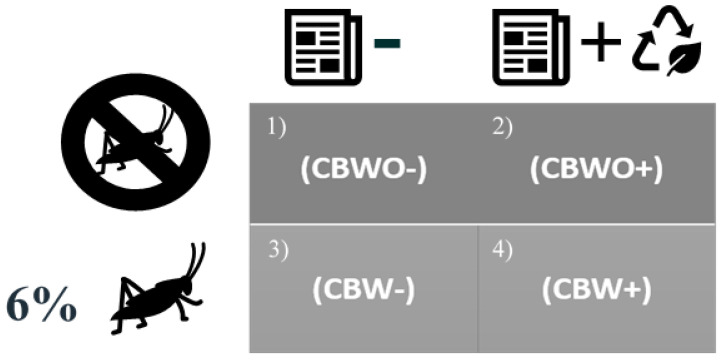
Treatments presented under informed conditions. **ECP:** Edible-cricket protein. **CB:** Chocolate brownies. **WO:** Without; **W:** With. **ECP−:** “No ECP” informed condition. **ECP+:** “Contains ECP + benefits” informed condition. **(1) CBWO−:** chocolate brownies without ECP (CBWO) presented under the ECP− informed condition; **(2) CBWO+:** CBWO presented under the ECP+ informed condition; **(3) CBW−:** chocolate brownies with 6% ECP (CBW) presented under the ECP− informed condition; **(4) CBW+:** CBW presented under the ECP+ informed condition.

Computer-based questionnaires were administered to panelists and their responses were collected using Qualtrics software (Qualtrics, Provo, UT, USA). Chocolate-brownie treatments (Figure 1) were presented simultaneously, and consumers were instructed to evaluate them in a monadic-sequential order following screen instructions, following a complete randomized block design.

**Figure 2:**
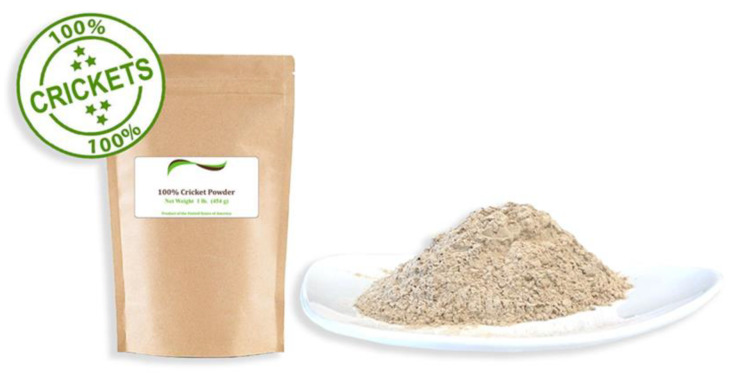
Edible cricket protein (ECP) picture presented in the ECP+ informed condition^†^. ^†^ Treatments are described in Figure 1.

Before evaluating a chocolate brownie (CB) sample without (WO) or with (W) edible cricket protein (ECP) (CBWO and CBW, respectively), an informed condition regarding the sample’s absence of ECP powder (ECP−: “please closely observe the brownie, this brownie does not contain ECP”) or the sample’s presence of ECP powder accompanied by ECP picture (Figure 2) and its nutritional and environmental benefits (ECP+: “please closely observe the brownie, this brownie contains ECP. Edible insects are safe to eat and are considered a sustainable source of high-quality protein and other nutrients. Edible insect production has less negative environmental impact than traditional livestock production. An estimated 2 billion people worldwide consume edible insects”) was disclosed to the subjects. Then, the participants: (1) rated their expected liking (before tasting) with a 9-point-hedonic scale (left-anchored dislike extremely and right-anchored like extremely) for appearance, aroma, texture, overall flavor, and overall liking (OL), (2) rated their actual liking (upon tasting) with the abovementioned scale for aroma, texture, overall flavor, and OL, and (3) expressed their willingness to consume and to purchase the sample if it were commercially available with a binomial scale (Yes or No) for each of the four treatments: CBWO− (no ECP in formulation presented with “no ECP” claim), CBWO+ (no ECP in formulation presented with “ECP+benefits” claim), CBW- (formulation with ECP presented with “no ECP” claim), and CBW+ (formulation with ECP presented with “ECP+benefits” claim).

### 2.3. Statistical Analysis

Data analyses were performed using the XLSTAT (Addinsoft, New York, NY, USA) statistical software version 2020 [26], R software version 4.0.3 (RStudio, Inc., Boston, MA, USA), and the Statistical Analysis Software (SAS) version 9.4 (Cary, NC, USA) with α = 0.05 significance level. Chocolate brownie treatments’ sensory evaluation by subjects followed a balanced and randomized block design (panelists as blocks) with a factorial arrangement (formulation and informed condition factors with two-way interactions). Multi-way analysis of variance (ANOVA) with a mixed-effects model (demographics, liking moment (expected and actual), formulation and informed condition effects with up to three-way interactions between gender, formulation and informed condition and between liking moment, formulation, and informed condition as fixed effects, having panelists as a random effect), and a post hoc Tukey’s HSD test (*p* < 0.05) were used to assess differences in the expected and actual hedonic ratings of the treatments. Principal Component Analysis (PCA) was used to elucidate the graphical relationship between treatments, expected and actual hedonic ratings. Agglomerative clustering analysis using the Euclidean-distance dissimilarity, Ward’s linkage, and average silhouette width to determine the ideal number of clusters was applied to segment consumers’ actual hedonic ratings into homogeneous liking profiles. Two-sided Cochran’s Q test followed by asymptotic McNemar test for post hoc multiple pairwise comparisons [27] with *p*-value adjusted by false discovery rate [28] was used to compare the frequencies of “likers” cluster across treatments segmented by gender. The proportion of “likers” across genders were compared with two-population proportions Z-tests. The same procedure (segmented by gender) was used to investigate if significant (*p* < 0.05) differences in consumption intent and purchase intent existed among the treatments and to compare the magnitude of the difference between consumption and purchase intent within treatments. The proportion of consumption and purchase intent across genders was compared for each treatment with two-population proportions Z-tests.

## 3. Results and Discussion

### 3.1. Significance of Effects on Sensory Acceptability

Sensory acceptability scores and results from analysis of variance (ANOVA) for the main and interaction effects are shown in Table 1, Table 2 and Table 3. The analysis of variance for the sensory liking variables (Table 1) showed that the gender effect (female or male) was only significant (*p* < 0.05) for texture liking and for overall flavor and overall liking (OL) when interacting with formulation (CBWO or CBW). In general, male participants reported higher texture liking scores than female participants (disregarding all other main effects, Table 3). The levels of gender influenced the way overall flavor and OL of treatments were evaluated depending on formulations. The formulation effect was significant (*p* < 0.05) for all sensory liking variables studied (Table 1). Disregarding the informed condition, liking moment, and the other main effects, CBWO obtained higher liking scores than CBW (CBWO = 6.65 vs. CBW = 6.38; CBWO = 6.72 vs. CBW = 6.52; CBWO = 6.41 vs. CBW = 5.98; CBWO = 6.61 vs. CBW = 6.23; CBWO = 6.60 vs. CBW = 6.26) for appearance, aroma, texture, overall flavor, and OL, respectively (data derived from Table 2). Liking moment (expected or actual) and its interaction with formulation were significant (*p* < 0.05) for texture liking, overall flavor, and OL. Disregarding all the other effects, liking moment affected the liking of the texture, overall flavor, and OL of the treatments depending on the formulation level. Informed condition (ECP− or ECP+) effect was only significant (*p* < 0.05) for aroma and overall flavor liking; however, its two-way interaction with liking moment was significant (*p* < 0.05) not only for aroma and overall flavor liking but also for texture and OL. For aroma and overall flavor liking of the treatments, ECP− informed condition led to higher liking scores than the ECP+ informed condition (ECP− = 6.68 vs. ECP+ = 6.55; ECP− = 6.49 vs. ECP+ = 6.36, respectively; data derived from Table 2). For all the sensory attributes studied (except appearance), liking moment altered the way subjects rated their liking for the treatments depending on the informed condition level. The two-way interaction between formulation and informed condition was only significant (*p* < 0.05) for aroma liking (Table 1).

### 3.2. Effects of Formulation and Edible Cricket Protein and Benefits Disclosure on Sensory Acceptability

**Figure 3:**
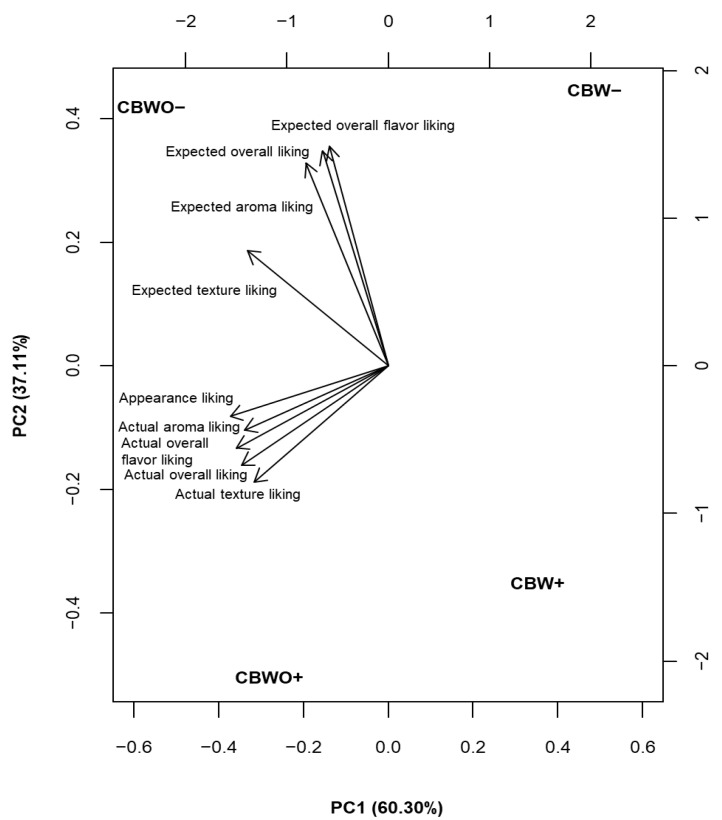
Principal component analysis (PCA) biplot visualizing expected and actual sensory liking and treatments ^†^. ^†^ Treatments are described in Figure 1.

Figure 3 depicts the separate contribution of formulation and informed condition effects to the observed variability across treatments in the sensory spectrum. The principal component 2 (explaining 37% of the observed variability among treatments) mainly represents the expected acceptability, and, to a lesser extent, expected texture acceptability, and is more influenced by informed condition effect than by formulation. Table 2 presents the least squares means for expected (before tasting) and actual (after tasting) attributes’ liking of treatments (Figure 1). When presenting formulations under the ECP− informed condition, appearance liking was higher for CBWO than for CBW, but when presenting them under the ECP+ informed condition, there was no difference across formulations, indicating a bias triggered by the informed condition and probably feelings driven by mental associations with entomophagy [29]. For either formulation (CBWO or CBW), expected flavor liking was higher (*p* < 0.05) for ECP− informed condition than for ECP+. Although the ECP+ informed condition contained information about environmental and nutritional benefits obtained from ECP consumption, it negatively impacted both brownie formulations’ (CBWO and CBW) expected flavor liking. Lammers et al. [30] found similar results when studying the willingness to consume an insect burger and buffalo worms by German consumers and reported that sustainability awareness was not an important driver for the willingness to consume insects.

However, for expected aroma and OL, the ECP+ informed condition negatively impacted only CBWO (Table 2). Expected liking did not differ (*p* > 0.05) across genders (Table 3).

Participants’ mindsets associated with food neophobia [31], disgust towards entomophagy [23], and other negative product-elicited emotions [32] may have contributed to the observed negative sensory-liking expectations for CBWO and flavor liking expectations for CBW when presented with ECP+ informed condition. Food neophobia and disgust emotion are the major mental constraints in the Western culture for the acceptability of entomophagy [33,34]. However, overcoming disgust emotion seems more challenging than prevailing over food neophobia because the familiarity of products containing insect protein can be improved through novel marketing campaigns such as advertising performed by “influencers” on social media platforms [30]. On the other hand, disgust emotion relies on existing associations between insects and other variables also considered disgusting, such as feces and decaying matter [35], which exert a higher predictive effect for the willingness to consume insects than neophobia [32].

Actual-liking scores were higher for CBWO than CBW for either informed condition (except for aroma when formulations were presented under ECP+ informed condition). Actual-liking scores were not significantly different between ECP− and ECP+ informed conditions for either formulation (CBWO and CBW; Table 2), showing a minimal effect of the informed conditions, which is also reflected in Figure 3 by the separation in terms of the principal component 1 (explaining 60% of the observed variability among treatments) mainly represented by formulation (CBWO− and CBWO+ on the left vs. CBW− and CBW+ on the right side). Schouteten et al. [36] reported similar findings when studying the effect of the informed conditions (blind vs. informed about the ingredient composition showing benefits and food safety for insect ingredient) using burgers formulated with insect, plant-based, and meat-based ingredients. Insect-based vs. meat burgers differed in their sensory profiles regardless of the condition in which they were evaluated; insect-based burgers required further product development to improve their sensory quality. In the present study, the only significant difference across genders was observed in the liking for overall flavor and OL, which was lower for females than for males for CBW− (Table 3), possibly because females exhibited higher taste sensitivity towards ECP or lower tolerance to changes in chocolate flavor than males in the brownie formulation.

Despite the apparent minimal effect of the informed conditions, it influenced the disconfirmation mechanism for CBW and CBWO, which affects the perception and liking of foods [37,38,39]. Anderson [40] proposed four psychological mechanisms to explain the effect on product evaluation and customer satisfaction upon disconfirmed expectancy on the perceived product performance: (1) assimilation, (2) contrast, (3) assimilation–contrast, and (4) generalized negativity. Assimilation theory hypothesizes that individuals will try to match the perception of a product with their expectations. Contrast theory supposes an increment of the real difference between the actual product and the expected product, resulting in under-rating or over-rating of products compared to a scenario without expectations for negative and positive disconfirmation, respectively. Assimilation–contrast theory assumes existing limits for acceptance and rejection of products upon their perception. When the discrepancy between the expectation and actual perception of an attribute is sufficiently large, the product falls into the rejection region, leading to the abovementioned contrast effect. Instead, if the experienced discrepancy is too small, the product evaluation will take place based on the assimilation theory. Generalized negativity theorizes that a generalized hedonic state will occur if any disconfirmation occurs, leading to lower product ratings than if it had matched with expectations.

In this study, the contrast effect dominated the participants’ evaluations when presenting CBW under ECP− informed condition, resulting in significant negative disconfirmation for all sensory attributes while for CBWO, significant negative disconfirmation was observed only for texture. The observed negative disconfirmation for CBW when participants were informed no ECP was present neither its benefits (ECP−) could be attributed to a “surprise effect”. Because consumers were informed that no ECP was present in the formulation, they expected regular chocolate brownie’s sensory properties and based their liking expectation on that piece of information and CBW− appearance. However, when noticing large differences in the sensory profile of CBW− compared to their expectations, a negative disconfirmation was driven possibly by the ECP “additional flavor” that occurred. On the other hand, the assimilation effect was more dominant when presenting both formulations (CBWO and CBW) under the ECP+ informed condition, resulting in non-significant disconfirmation because participants’ mindset was already conditioned to taste something that they might or might not like.

Tan, Fischer, van Trijp and Stieger [33] reported higher sensory acceptability of novel foods, including insect-based foods after tasting compared to before tasting. Novel-food products such as chocolate brownies with ECP could benefit from attributes exhibiting no significant negative disconfirmation and positive disconfirmation (even if not significant). A mean liking score of 7 or higher on a nine-point hedonic scale is usually indicative of highly acceptable sensory quality [41], but considering the introduction of ECP to human diet represents a new concept for American consumers, CBW+ overall acceptability (actual OL = 6.17) seems a promising scenario for ECP incorporation into chocolate brownies whose formulation is yet to be optimized.

### 3.3. Overall Differences in Sensory Acceptability Segmented by Gender

**Figure 4:**
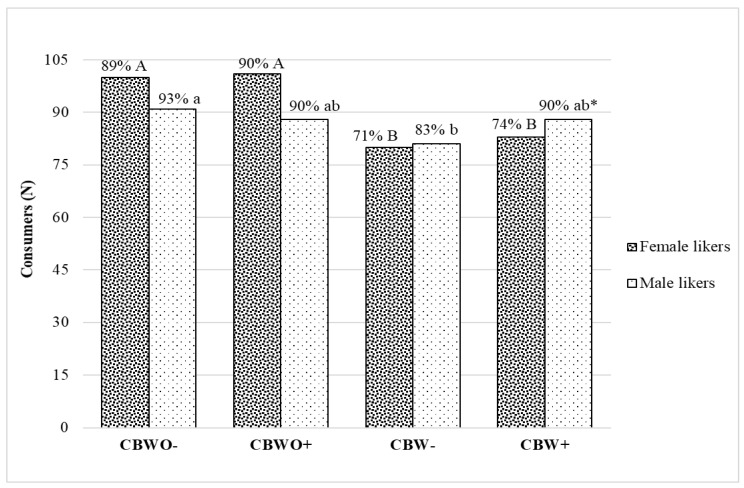
Likers and dislikers gender frequency plot across treatments ^†^. Data are frequencies of *n* = 112 female and *n* = 98 male consumers analyzed by Cochran’s Q test with asymptotic McNemar test for post hoc multiple pairwise comparisons and *p*-value adjustment by false discovery rate. Liking clusters were determined through agglomerative clustering analysis from actual hedonic attributes rating applying Euclidean-distance dissimilarity, Ward’s agglomeration method, and average silhouette width to determine the ideal number of clusters. ^†^ Treatments are described in Figure 1. Uppercase/lowercase letters represent a significant (*p* < 0.05) difference in the proportion of “likers” across treatments within female/male consumers. * Significant (*p* < 0.05) difference in “likers” proportion across genders (Z-test 2 population proportions).

Dimensions of actual liking were evaluated at the multivariate level jointly by clustering the panelists’ actual liking for the treatments (Figure 4). Agglomerative clustering based on Euclidean distance dissimilarity using Ward’s agglomeration method and average silhouette width to obtain the ideal number of clusters yielded two profiles for the subjects who evaluated the treatments, “dislikers” and “likers”. The frequency of “likers” was compared across treatments based on gender. For females, the formulation was the leading factor for acceptability of the treatments while the informed condition was not. A significantly (*p* < 0.05) higher proportion of female likers for treatments without ECP (CBWO− = 89% and CBWO+ = 90%) vs. those with ECP (CBW− = 71% and CBW+ = 74%) was observed.

On the contrary, the proportion of male likers was comparable across treatments without ECP (CBWO− and CBWO+) and CBW+, and treatments containing ECP (CBW− and CBW+) presented a similar proportion of likers to CBWO+. These findings suggest a positive effect of the informed condition on the acceptability of chocolate brownies with ECP for male consumers.

Evidence of gender effect on the acceptability of edible insects is variable [42]. Some have found significant effects of gender on the acceptability of food containing edible insects [25,34,43,44] while others have not [45,46]. A study by Megido, Gierts, Blecker, Brostaux, Haubruge, Alabi and Francis [25] with Belgian students (18–25 years old) reported a significant effect of gender (males exhibited less neophobic behavior than women) in addition to familiarity with edible insects, product appearance, and taste on the overall liking of hybrid insect-based burgers. Similarly, in a cross-sectional study with Belgian non-vegetarian subjects involved in food purchase, Verbeke [34] found that males were more willing to incorporate edible insects into their diets than females, and individuals who wished to reduce meat consumption had a strong orientation toward convenience foods and were concerned about the environmental impact of their food choices. On the other hand, consumers who enjoyed the taste of meat and were convinced of its health benefits were less likely to incorporate edible insects into their diets. In the online survey study about attitudes toward food conducted by Ruby, Rozin and Chan [43], men’s readiness to taste edible insects was higher than for females, mainly in the U.S. Disgust emotion, notions of benefits, sensation seeking, and pleasure of telling others about consumption of unusual foods were significant parameters for the prediction of edible insects’ acceptability.

### 3.4. Consumption and Purchase Intent Segmented by Gender

**Figure 5:**
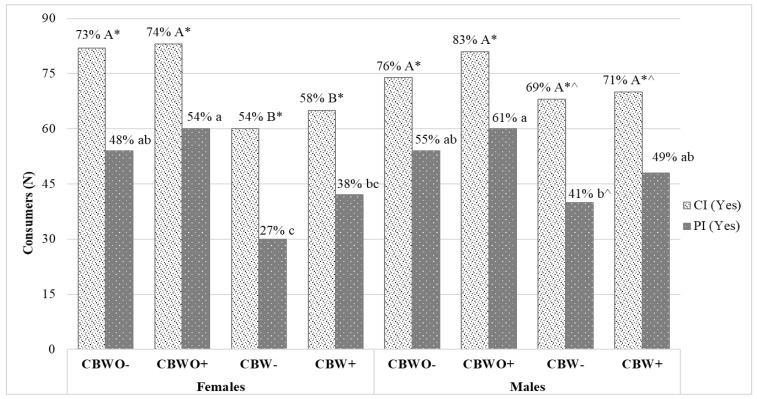
Gender’s consumption intent and purchase intent plot across treatments ^†^. Data are frequencies of “Yes” responses per treatment from *n* = 112 female and *n* = 98 male consumers analyzed by Cochran’s Q test with asymptotic McNemar test for post hoc multiple pairwise comparisons and *p*-value adjustment by false discovery rate. ^†^ Treatments are described in Figure 1. Uppercase/lowercase letters represent significantly (*p* < 0.05) different consumption intent/purchase intent frequencies across treatments for each gender. * Significant (*p* < 0.05) difference across purchase intent and consumption intent within treatments for each gender. ^ Significant (*p* < 0.05) difference in consumption intent/purchase intent frequencies across genders (Z-test 2 population proportions) within treatments.

The frequency distribution for consumption and purchase intent by gender is shown in Figure 5. For females, a similar scenario to overall product sensory acceptability described above was observed. Treatments containing ECP (CBW− and CBW+) had a lower (*p* < 0.05) frequency of consumption intent than those without ECP (CBWO− and CBWO+) regardless of the informed condition. On the contrary, males had a similar frequency of consumption intent across all treatments. Females may have had lower thresholds for the detection or recognition of ECP in the brownie formulation than males [47] or male consumers while recognizing a difference in the sensory properties of treatments with ECP, but still presented the same willingness to consume them as for those without ECP because males had higher blind acceptability for ECP than women [48,49,50].

Regarding purchase intent, females exhibited higher (*p* < 0.05) frequency for treatments without ECP (CBWO− and CBWO+), but the treatment containing ECP and the informed condition (CBW+) achieved similar purchase intent to CBWO−, demonstrating a positive effect of the informed condition (purchase intent increased from 27% for CBW− to 38% for CBW+; Figure 5). For males, the purchase intent frequency was significantly lower only for CBW− when compared to CBWO+. In this scenario, the informed condition (ECP+) in CBW allowed CBW+ to achieve comparable purchase intent to that of CBWO− and CBWO+. This suggests that there is a greater positive effect of the informed condition towards the willingness to pay for brownies containing ECP in males than in females [51]. Drivers for this behavior in males may include increased sensation-seeking, which is related to an individual’s disinhibition, experience-seeking, susceptibility to boredom, and tendency to seek thrill and adventure [43] or experiencing less disgust [52]. These results agree with the aforementioned section regarding males’ overall product sensory acceptability. However, all treatments presented a significant (*p* < 0.05) discrepancy in the frequency of consumption intent vs. purchase intent, with the consumption intent proportion higher. This behavior could be partly explained by the need for improvement in terms of formulation for all brownies, not only those containing ECP [53]. This may suggest that consumers may be ready to experience tasting products formulated with edible insects [23] but are not necessarily willing to pay for them as this involves the consumers’ perceived importance of the ECP benefits and their socioeconomic status.

Some studies report a significant effect of communicating benefits about entomophagy towards improving edible insects’ acceptability and participants’ willingness to taste [35,43,51,54,55], which is dependent on the subjects’ degree of environmental consciousness [34,56,57,58,59,60], while others found it insufficient to promote their acceptability [46,50,61,62]. However, a consumption intent above 50% and a purchase intent of 49% achieved by CBW+ among males offer a promising scenario when still being in the introductory stage for this kind of novel product.

## 4. Conclusions and Future Studies

This research investigated consumers’ hedonic perceptions, consumption, and purchase behaviors towards ECP contained in familiar chocolate brownies. The acceptability of ECP in chocolate brownies differed across genders, with males being more likely to consume and purchase chocolate brownies containing ECP than females. Although formulation affected actual liking of chocolate brownies more than the informed condition, the latter prevented significant negative disconfirmation for all sensory attributes in chocolate brownies containing ECP. The actual OL obtained for CBW+ (regardless of gender) suggests a promising scenario for the incorporation of ECP into similar products as well as products consumers are willing to try. Moreover, informing consumers about the presence and nutritional and environmental benefits of ECP (ECP+) positively impacted the purchase intent of CBW among females, while among males it decreased the sensory liking differences among formulations favoring the proportion of likers for CBW. As consumption of edible insects among Westerners is very limited, information regarding edible insects and their consumption, including safety, environmental impact, and nutritional benefits may improve familiarity and alleviate aversion to entomophagy. Therefore, the findings from this study may be helpful to guide future development of products incorporated with ECP for the Westerner diets.

For future studies, differences between samples regarding texture and flavor should be further investigated and characterized either with a traditional descriptive analysis with trained panelists or with novel rapid methods such as a consumer based CATA descriptive panel [63]. Once key sensory attributes have been identified in brownies containing ECP, another consumer study involving the intensity perception of such attributes could be conducted to determine which are responsible for the observed differences in acceptability across formulations. Evaluating different ECP suppliers through a descriptive profile is highly recommended to identify key attributes and their ideal levels through consumer rejection threshold studies. Lastly, evaluating the emotional profile of treatments in a complementary way to hedonic discrimination to identify drivers of liking and important product-elicited emotions that predict acceptability, consumption, and purchase behavior is recommended.

## Figures and Tables

**Table 1:** ANOVA ^†^ table for sensory acceptability ^‡^ of treatments ^§^.

Effects	Appearance *		Aroma		Texture		Overall Flavor		Overall Liking	
	F Value	P_r_ > F	F Value	P_r_ > F	F Value	P_r_ > F	F Value	P_r_ > F	F Value	P_r_ > F
Gender	1.54	0.22	0.01	0.91	4.33	0.04	3.49	0.06	2.54	0.11
Liking moment	-	-	0.51	0.48	15.46	<0.01	13.22	<0.01	17.95	<0.01
Formulation	39.34	<0.01	13.38	<0.01	50.79	<0.01	39.64	<0.01	35.00	<0.01
Informed condition	0.79	0.37	*5.78*	*0.02*	0.23	0.63	4.67	0.03	3.07	0.08
Liking moment*Formulation	-	-	0.80	0.37	6.57	0.01	19.95	<0.01	15.58	<0.01
Liking moment*Informed condition	-	-	14.40	<0.01	13.96	<0.01	26.81	<0.01	29.10	<0.01
Formulation*Informed condition	2.79	0.10	*4.09*	*0.04*	0.01	0.93	1.81	0.18	2.84	0.09
Gender*Formulation	0.04	0.84	1.61	0.20	1.31	0.25	6.64	0.01	6.32	0.01
Gender*Informed condition	0.03	0.87	0.47	0.49	0.03	0.85	0.21	0.65	0.35	0.55
Liking moment*Formulation*Informed condition	-	-	1.25	0.26	1.00	0.32	0.22	0.64	0.00	0.98
Gender*Formulation*Informed condition	0.11	0.74	1.14	0.29	0.00	0.96	0.25	0.62	0.16	0.69

^†^ ANOVA = Analysis of variance 2 genders (female and male), 2 levels of liking moment (expected and actual), 2 formulations (CBWO and CBW), and 2 informed conditions (ECP− and ECP+). ^‡^ Liking data from *n* = 210 consumers were collected using a 9-point hedonic scale (1 = dislike extremely, 9 = like extremely) and analyzed by a mixed-effects model with panelists as a random effect. ^§^ Treatments are described in Figure 1. * Appearance liking determined only before taste.

**Table 2:** Expected and actual sensory acceptability ^†^ of treatments ^‡^.

Treatments	CBWO−		CBWO+		CBW−		CBW+		SEM ^§^
Likings	Expected	Actual	Expected	Actual	Expected	Actual	Expected	Actual	
Appearance	6.67^A^		6.64^A^		6.33^B^		6.44^AB^		0.28
Aroma	6.90^A^	6.77^AB^	6.52^BCD^	6.67^ABCD^	6.70^ABC^	6.36^D^	6.42^CD^	6.60^ABCD^	0.25
Texture	6.58^A^	6.21^BCD^	6.33^ABC^	6.53^AB^	6.25^ABC^	5.68^E^	6.10^CD^	5.87^DE^	0.31
Overall Flavor	6.83^A^	6.60^ABC^	6.34^BCD^	6.67^AB^	6.67^AB^	5.84^E^	6.28^CD^	6.13^DE^	0.28
Overall Liking	6.86^A^	6.53^AB^	6.35^BC^	6.65^AB^	6.65^AB^	5.87^D^	6.33^BC^	6.17^CD^	0.28

^†^ Liking data are least-squares means from *n* = 210 consumers. Different uppercase letters within a row represent significant (*p* < 0.05) differences across treatments (Tukey’s means separation) for each attribute. ^‡^ Treatments are described in Figure 1. ^§^ Standard error of the least square means.

**Table 3:** Sensory acceptability ^†^ of treatments ^‡^ by gender.

Moment	Treatments	CBWO−		CBWO+		CBW−		CBW+		SEM ^§^	
	Gender	Female	Male	Female	Male	Female	Male	Female	Male	Female	Male
Expected (before tasting)	Appearance	6.56^A^	6.78^A^	6.54^A^	6.74^A^	6.24^A^	6.41^A^	6.33^A^	6.54^A^	0.30	0.31
	Aroma	7.10^AC^	6.96^ABE^	6.64^BDEF^	6.65^ABCDEF^	6.90^ABCD^	6.74^ABCDEF^	6.54^EF^	6.55^CDF^	0.27	0.28
	Texture	6.49^A^	6.73^A^	6.21^AB^	6.50^AB^	6.16^AB^	6.38^AB^	5.89^B^	6.38^AB^	0.31	0.32
	Overall Flavor	6.74^AB^	6.89^A^	6.26^C^	6.38^BC^	6.57^ABC^	6.71^ABC^	6.18^C^	6.33^BC^	0.28	0.29
	Overall Liking	6.83^AB^	6.93^A^	6.34^C^	6.40^BC^	6.61^ABC^	6.71^ABC^	6.27^C^	6.43^BC^	0.28	0.29
Actual (after tasting)	Aroma	6.75^A^	6.53^AB^	6.54^AB^	6.55^AB^	6.09^B^	6.39^AB^	6.45^AB^	6.50^AB^	0.30	0.31
	Texture	6.00^ABC^	6.36^ABC^	6.37^AB^	6.63^A^	5.34^D^	5.98^BCD^	5.67^CD^	6.01^BCD^	0.37	0.38
	Overall Flavor	6.57^A^	6.67^A^	6.62^A^	6.77^A^	5.44^C^	6.31^AB^	5.85^BC^	6.47^AB^	0.34	0.35
	Overall Liking	6.45^A^	6.58^AB^	6.56^A^	6.69^A^	5.46^C^	6.26^AB^	5.90^BC^	6.41^AB^	0.34	0.36

^†^ Liking data are least-squares means from *n* = 112 female and *n* = 98 male consumers. Different uppercase letters within a row represent significant (*p* < 0.05) differences across treatments and gender (Tukey’s means separation) for each attribute. ^‡^ Treatments are described in Figure 1. ^§^ Standard error of the least square means.

## Data Availability

The data that support the findings of this study are available from the corresponding author upon reasonable request.
